# TGF-β Signaling in Microglia: A Key Regulator of Development, Homeostasis and Reactivity

**DOI:** 10.3390/biomedicines12112468

**Published:** 2024-10-28

**Authors:** Lulin Li, Bryan Sun, Odette A. Harris, Jian Luo

**Affiliations:** 1 Palo Alto Veterans Institute for Research, VA Palo Alto Health Care System, Palo Alto, CA 94304, USA; 2 Department of Neurosurgery, Stanford University School of Medicine, Stanford, CA 94305, USA; 3 Polytrauma System of Care, VA Palo Alto Health Care System, Palo Alto, CA 94304, USA

**Keywords:** microglia, reactive microglia, TGF-β, homeostasis, Alzheimer’s disease, APOE

## Abstract

Microglia, the resident immune cells of the central nervous system (CNS), are crucial for normal brain development and function. They become reactive in response to brain injury and disease, a process known as microglial reactivity. This reactivity, along with microglial homeostasis, is tightly regulated by the local microenvironment and interactions with surrounding cells. The TGF-β signaling pathway plays an essential role in this regulation. Recent genetic studies employing microglia-specific manipulation of the TGF-β signaling pathway have shed light on its significance in microglial development, homeostasis and reactivity. This review provides an updated overview of how TGF-β signaling modulates microglial function and reactivity, contributing to our understanding of microglial biology in health and disease.

## 1. Introduction

Microglia, the resident immune cells of the brain, are essential for maintaining CNS homeostasis, responding to injury and orchestrating immune responses [1]. Transforming Growth Factor-beta (TGF-β) is a key regulator of microglial function in the adult central nervous system (CNS) [2]. TGF-β signaling is pivotal in maintaining microglia in a homeostatic state under normal conditions. This cytokine not only preserves microglial identity but also modulates its responsiveness to environmental changes, balancing their roles in neuroprotection and immune activation. In pathological conditions, TGF-β can regulate microglial reactivity, potentially exacerbating or mitigating CNS damage depending on the context. This review summarizes recent findings from genetic studies on microglial TGF-β signaling in adult mice, emphasizing its importance in preserving neural homeostasis and its involvement in CNS disease development.

## 2. TGF-β Signaling in the Brain

TGF-β is a pleiotropic cytokine with a wide range of essential functions on various cell types throughout the body [3]. In the brain, TGF-β regulates multiple aspects of brain function including maintaining cellular homeostasis, regulating immune responses and modulating the activity of neurons and glial cells [4]. The intricate regulation of TGF-β signaling is essential for proper brain function and aberrant TGF-β signaling contributes to the pathogenesis of neurological disorders [5].

TGF-β is secreted in a biologically inactive (latent) form as part of a latent complex. Before binding to its receptors, latent TGF-β must be activated. Activators of latent TGF-β include cell surface integrins (αvβ6 and αvβ8), as well as various proteases [3]. Notably, in some instances, αvβ8-mediated autocrine signaling of TGF-β can occur without the release of TGF-β1 from its latent form [6].

**Figure 1:**
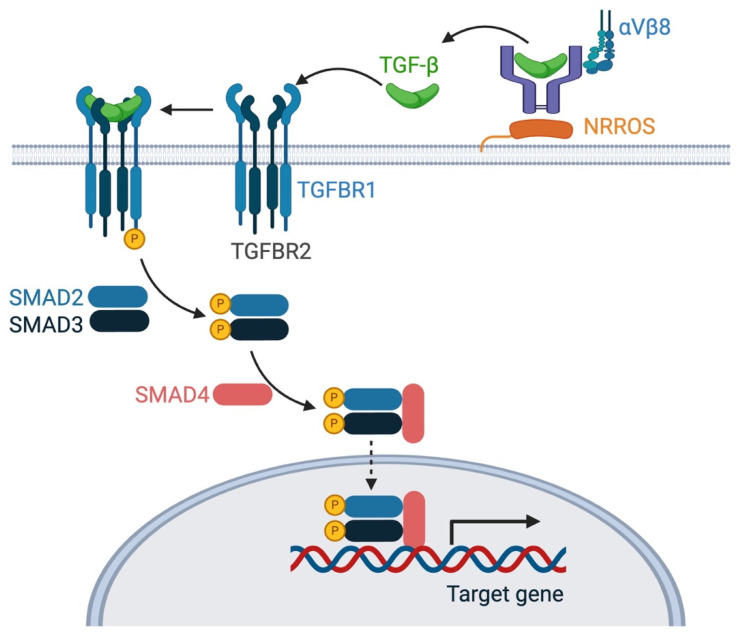
TGF-β activation and signaling in microglia. TGF-β is produced in a latent form as part of a latent complex tethered to the cell surface via NRROS (also known as LRRC33). The latent complex consists of mature dimeric TGF-β, associated with latency-associated peptide and latent TGF-β-binding protein. Latent TGF-β can be activated and released from the complex by integrins (αvβ8) and proteases. Active TGF-β binds to its receptors and initiates SMAD signaling to exert its biological effects. This figure was created using BioRender (https://biorender.com/ (accessed on 14 October 2024)).

TGF-β exerts its effects through binding to TGF-β receptor types 1 and 2 (TGFBR1/2) on the surface of target cells [3]. Receptor binding initiates a signaling cascade that involves the phosphorylation of receptor-regulated SMAD proteins (SMAD2/3). Phosphorylated SMAD2/3 then form complexes with SMAD4, translocate to the nucleus and regulate the expression of TGF-β/Smad-responsive genes (Figure 1). In the CNS, TGF-β and its receptors are expressed in a region- and cell-specific manner. Consequently, TGF-β signaling is finely tuned, with tight regulation ensuring that its effects are cell type- and context-dependent.

During CNS development, TGF-β plays a crucial role in neurogenesis, neuronal migration, gliogenesis, angiogenesis and the formation of the blood–brain barrier (BBB) [7]. It is involved in neuron maturation, guiding axonal growth and ensuring the proper formation of synaptic connections [4]. Beyond development, TGF-β continues to influence synaptic plasticity, a process essential for learning and memory [7]. In adult mice, TGF-β signaling promotes hippocampal long-term potentiation (LTP), particularly the transition from early to late-phase LTP [8,9]. TGF-β signaling also supports adult neurogenesis in the dentate gyrus and keeps the balance of proliferation versus maturation in differentiating immature cells [10]. Smad2 deficiency blocks CX3CL1-mediated neurogenesis [11] and specifically compromises spatial learning in mice [10], suggesting that Smad2 participates in adult neural plasticity. Disrupted TGF-β signaling and hippocampal dysfunction contribute to neurodevelopmental and behavioral disorders associated with USP9X variants [12].

In the context of CNS injury, such as traumatic brain injury (TBI) or stroke, TGF-β plays a neuroprotective role by promoting the survival of neurons and limiting the extent of damage [5]. It does so by modulating the inflammatory response, enhancing the clearance of cellular debris and supporting the repair of damaged tissue. TGF-β also influences the reactivity of astrocytes [5,7], another type of glial cell, which contributes to the formation of the glial scar-a structure that, while preventing further damage, can also inhibit axonal regeneration.

Alterations in TGF-β signaling have been implicated in the pathogenesis of several neurodegenerative diseases, including Alzheimer’s disease (AD), Parkinson’s disease (PD) and amyotrophic lateral sclerosis (ALS) [5]. In AD, for example, dysregulated TGF-β signaling has been associated with increased amyloid-beta (Aβ) deposition, tau pathology and chronic neuroinflammation, all of which contribute to disease progression [13]. Similarly, in PD and ALS, aberrant TGF-β signaling can exacerbate neuroinflammation and neuronal loss [5]. Understanding these mechanisms is critical for developing therapeutic strategies that target TGF-β pathways to slow or prevent neurodegeneration.

Cognitive functioning is essential for achieving successful health and aging. Cognitive decline is a hallmark of many neurodegenerative diseases and negatively impacts quality of life. TGF-β signaling plays a crucial role in cognitive function by influencing synaptogenesis and plasticity, as well as the structure and function of the neurovascular unit [14]. TGF-β1 is implicated in cognitive deficits across various neurological diseases, including AD, cerebrovascular disease (CSVD), schizophrenia and depression, affecting multiple cognitive domains [14]. Target TGF-β signaling improves cognitive function [15].

In summary, TGF-β is a versatile cytokine with significant roles in the brain, from development and synaptic plasticity to neuroprotection and the regulation of neuroinflammation. Its ability to maintain homeostasis while modulating the brain’s response to injury and disease underscores its importance in CNS function. However, the complexity of TGF-β signaling also means that its dysregulation can contribute to the development and progression of neurodegenerative diseases. Ongoing research into TGF-β‘s diverse roles in the brain holds promise for novel therapeutic approaches aimed at harnessing its protective effects while mitigating its potential to drive pathology.

## 3. Microglia: Development, Homeostasis and Reactivity

Microglia, the resident innate immune cells of the CNS, are highly dynamic and plastic cells [16]. They play crucial roles under normal physiological conditions, including brain maturation and the maintenance of homeostasis, and are also critical players in injury, infection and disease.

Microglia originate from yolk sac progenitors during early embryonic development (reviewed in [17]). During embryogenesis, these progenitors migrate into the developing brain around embryonic day 9.5 in mice and proliferate, establishing the microglial population before the BBB fully forms. This early migration and proliferation ensure that microglia are well-positioned to participate in brain development, synaptic pruning and the establishment of the brain’s immune environment [18]. Once established, microglia self-renew and maintain their population into adulthood, independent of contribution from bone marrow-derived cells under normal physiological conditions [16]. This autonomy is a defining feature of microglia, ensuring a stable and consistent immune presence in the CNS throughout life. Interestingly, microglia in embryonic and early postnatal CNS differ from their adult counterparts, displaying an activated phagocytic state alongside enhanced proliferation and reactivity [19]. The transcriptional transition of fetal to adult microglia relies on a population of CD4+ T cells [20].

Aside from microglia, which reside in the parenchyma, brain macrophages also include non-parenchymal border-associated macrophages (BAMs) [21]. BAMs are primarily located in the choroid plexus, meningeal and perivascular spaces. Like microglia, BAMs originate from the yolk sac and infiltrate the brain early during embryogenesis; however, they express different surface markers than microglia [21]. BAMs play a variety of roles in the CNS, significantly influencing the cerebral immune environment and the development of cognitive decline associated with vascular and amyloid-related dementia [22]. During neuroinflammation, BAMs enter reactive states [23]. In an α-synuclein model of Parkinson’s disease, BAMs are the primary macrophage population, facilitating T cell recruitment and activation [24]. Additionally, in a mouse model of salt-sensitive hypertension, BAMs mediated cognitive impairment [25].

The homeostatic state of microglia is characterized by the low expression of pro-inflammatory genes and the high expression of genes associated with surveillance and repair functions. In a healthy brain, microglia exist in a ramified, ‘resting’ state, constantly extending and retracting their processes to monitor the microenvironment. This surveillance function is essential for detecting subtle changes in the CNS, such as the presence of apoptotic cells, synaptic changes, or minor injuries [2,26]. In their homeostatic state, microglia continuously monitor the CNS environment, removing debris, modulating synaptic activity and responding to injury or disease [18]. Microglial homeostasis is tightly regulated to balance their roles in surveillance, their support of neuronal function and their response to injury or infection [26]. This balance is influenced by various endogenous and external factors, including signals from neurons, astrocytes and neural precursor cells [27]. The critical role of homeostatic microglia in normal brain function has been highlighted by two recent studies showing that the absence of homeostatic microglia results in severe neuropathology, while the transplantation of healthy microglia can reverse these conditions [28,29].

Microglial reactivity is a hallmark of the CNS response to injury, infection and neurodegenerative diseases. Microglia are highly versatile and can rapidly respond to even subtle changes in CNS homeostasis [18]. Upon detecting changes in homeostasis or danger signals, microglia initiate a reactive program, transitioning through multiple reactive states, ultimately shifting into a phagocytic amoeboid macrophage phenotype [1]. This transformation is marked by alterations in gene expression, morphology and function, allowing microglia to respond swiftly to potential threats. Microglial reactivity is not a binary process but rather a spectrum of states, ranging from mild reactivity, characterized by increased surveillance and cytokine production, to full-blown reactivity, involving phagocytosis, the production of reactive oxygen species and the secretion of pro-inflammatory cytokines [1,16,18]. The specific microglial response can be either adaptive or maladaptive, depending on the nature and duration of the stimulus, leading to different outcomes in the context of trauma, injury, infection and stress [1,16]. While microglial reactivity is essential for CNS defense, chronic or excessive reactivity can drive neuroinflammation and contribute to the pathogenesis of neurodegenerative diseases such as AD, PD and multiple sclerosis (MS). In these conditions, microglia can shift into a maladaptive state, characterized by the release of pro-inflammatory cytokines, increased oxidative stress and the promotion of neurodegeneration [30]. In support of this, risk genes identified from genome-wide association studies for neurological and psychiatric disorders are primarily expressed in microglia and act through microglia [31].

Recent studies utilizing single-cell RNA sequencing (scRNA-seq) and single-nucleus RNA sequencing (snRNA-seq) have uncovered a spectrum of reactive microglial cell states as the brain ages and in age-related neurodegenerative conditions [16]. Various transcriptomically distinct states have been described, often characterized by how their genetic signatures differ from those of adult homeostatic microglia across different brain regions and over time. These reactive states include disease-associated microglia (DAM) [32], microglial neurodegenerative phenotypes (MGnDs) [33,34], activated response microglia (ARM) [35], lipid droplet-accumulating microglia (LDAM) [36], human AD microglia (HAMs) [37], white matter-associated microglia (WAM) [38], disease inflammatory macrophages (DIMs) [39] and terminally inflammatory microglia (TIM) [40]. These reactive microglial states increase in number with age, associated with the reduced expression of homeostatic genes, along with the increased expression of genes associated with endocytosis, lysosomal/phagocytic pathways and the regulation of immune responses [41]. The DAM [42] and MGnD [33,34] populations exhibit neuroprotective properties, while DIMs, LDAM and WAM represent dysfunctional and pro-inflammatory states and likely contribute to maladaptive responses to aging, neurodegeneration and functional decline. However, it should be noted that these states are dynamic and highly context-dependent, shifting at different stages of disease progression [43]. The same microglial states that are beneficial in certain contexts may become detrimental in others, depending on the complex interactions between microglia and their surrounding environment [16].

The different microglial states discovered by transcriptomics highlight significant advances in our understanding of microglia heterogeneity and their responses to disease. However, it is clear that mRNA expression does not consistently correlate with protein expression [44], emphasizing the need for proteomic analysis to gain deeper insights into microglial biology [45]. Several studies have conducted proteomic analyses of microglia in both human and mouse models of neurodegenerative diseases. These findings complement transcriptomic analyses, offering a more comprehensive understanding of microglial functions in the context of neurodegenerative disorders [46]. Proteomic profiling of human myeloid cells derived from blood, CSF and the brain using mass cytometry via time of flight (CyTOF) confirmed key transcriptomic signatures of human microglia at the protein level and microglia heterogeneity across different brain regions [47]. A recent meta-analysis of microglia from four quantitative mouse proteomics studies involving models of Alzheimer’s disease, LPS-induced inflammation and aging revealed a “reactive” state, characterized by the decreased expression of homeostatic markers and the upregulation of antigen-presenting and interferon response proteins. [45].

## 4. TGF-β Signaling in Microglia Development

TGF-β signaling is a major pathway regulating microglial development and maturation [17]. Early genetic knockout studies have highlighted its significance [2,4] and recent microglia-specific deletions further demonstrate that TGF-β signaling regulates microglia proliferation during embryonic development [48] in a SMAD-dependent manner [49,50]. The genetic deletion of Tgfbr2 (between E10.5 and E16.5) in hematopoietic cells in *Vav1*^iCre^*Tgfbr2*^fl/fl^ mice results in a reduced number of microglia in the brain throughout development [48]. In these mice, the remaining microglia exhibit an amoeboid morphology, characteristic of reactive or immature microglia and an altered phenotype, including the upregulation of CD45 and F4/80. However, there was no change in the number of BAMs [48], suggesting that TGF-β signaling is required for microglia development but not for BAMs. scRNA-seq analysis supports this idea. The construction of gene ontology networks from differentially expressed genes between BAMs and microglia reveals that microglia-enriched genes are associated with TGF-β signaling, while BAM genes are linked to blood vessel development, lipid and cholesterol metabolism, immune response and antigen presentation [51]. The transcription factor SMAD4, a downstream component of the TGF-β signaling pathway, is critical for driving microglial transcriptional signatures and inducing target genes such as SALL1 [49,50]. The deletion of SMAD4 in microglia leads to developmental arrest, resulting in microglia acquiring a BAM specification signature and causing memory impairment [49]. Functional interactions between SALL1 and SMAD4 are required for microglia-specific gene expression. SMAD4 directly binds to the Sall1 super-enhancer and is essential for Sall1 expression. In turn, SALL1 enhances SMAD4 binding and activity at microglia-specific enhancers [50]. The key role of TGF-β signaling in microglial development is also supported by the findings that the TGF-β-dependent transcription factor SALL1 plays a crucial role in guiding developmental microglia towards a homeostatic profile [51,52,53]. SALL1 expression is highly restricted to microglia [51,52]. SALL1-mediated transcriptional control silences an inflammatory program and preserves microglial identity in vivo. The inactivation of the murine Sall1 locus in vivo results in the conversion of microglia from homeostatic tissue macrophages to inflammatory phagocytes, leading to altered neurogenesis and disrupted tissue homeostasis [52].

The significance of TGF-β signaling is also evident in the development and maturation of human microglia. The analysis of published human scRNA-seq datasets reveals the consistent enrichment of Tgfb1 gene expression in human microglia across multiple independent studies [54]. In postnatal human microglia, TGF-β signaling maintains a homeostatic phenotype through SALL1 [55] via SMAD4- and SMAD2-dependent pathways [56]. Collectively, studies in both mice and humans have identified TGF-β as a key environmental cue that mediates the transition from yolk sac progenitors to parenchymal microglia [17].

The temporal and spatial patterns of microglial colonization support that microglia play a role in the early stages of brain development, including cell proliferation, differentiation and neovascularization [57], with TGF-β being a key factor in these processes. Microglia utilize TGF-β to interact with brain endothelial cells, thereby regulating endothelial cell heterogeneity [58] and vascular architecture and tissue mechanics [59].

## 5. TGF-β Signaling in Microglia Homeostasis

TGF-β signaling is critical for maintaining the homeostatic state of microglia. The role of TGF-β in microglial homeostasis is multifaceted, influencing gene expression, signaling pathways and cellular functions that are essential for the balanced activity of these cells within the CNS. This signaling pathway operates primarily through the TGF-β receptors on microglial surfaces, activating the SMAD-dependent and SMAD-independent pathways that regulate the transcription of key genes involved in microglial function. In particular, the SMAD-dependent pathway is known to be essential for the transcriptional regulation of genes that maintain microglial identity [49,50]. This mechanism helps microglia avoid unnecessary inflammatory responses that could harm the CNS.

Under physiological conditions, TGF-β maintains microglia in a surveillant state, enabling them to monitor the CNS environment for signs of injury or infection without triggering unnecessary inflammatory responses. Tgfbr1 is a key component of the “sensome”, the mechanisms microglia utilize to sense and respond to environmental stimuli [60]. TGF-β signaling in microglia modulates the expression of key genes that define their identity and function [41,53]. This signaling pathway ensures that microglia perform their protective functions without inducing harmful inflammation, thereby preserving CNS homeostasis. Interestingly, microglia appear more sensitive to TGF-β deficiency compared to other brain cells. The conditional deletion of integrin αVβ8 (necessary for activating latent TGF-β, see Figure 1) specifically from all the CNS neuroepithelial lineage cells using nestinCre (*Itgb8ΔCNS* mice), reduces active TGF-β levels in the CNS [61]. This reduction leads to diminished TGF-β signaling mainly in microglia, but not in other cell types. Adult microglia isolated from *Itgb8ΔCNS* mice displayed a gene expression profile nearly identical to that observed in TGF-β1 knockout mice [61].

**Figure 2:**
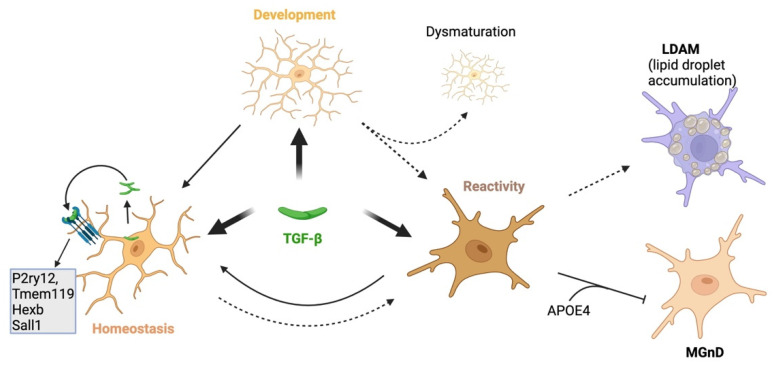
TGF-β plays a crucial role in microglial development, homeostasis and reactivity. During embryonic development, TGF-β1 induces microglial maturation. Deficiencies in TGF-β signaling during this stage lead to dysmaturation and increased reactivity. In adult mice, TGF-β signaling is pivotal for maintaining microglial homeostasis and preventing reactivity under physiological conditions. Deficient TGF-β signaling in mature microglia alters homeostatic gene expression, resulting in a reactive phenotype. In reactive states, deficient TGF-β signaling promotes lipid droplet accumulation (LDAM), driving microglia toward a pro-inflammatory phenotype. In contrast, increased TGF-β signaling contributes to APOE4-induced defective microglial responses (blunted MGnD response). Dashed lines illustrate pathways influenced by deficient TGF-β signaling, while solid lines represent normal or increased TGF-β signaling. This figure was created using BioRender (https://biorender.com/ (accessed on 14 October 2024)).

Gene profiling and quantitative mass spectrometry analyses have revealed a unique microglial signature that is dependent on TGF-β signaling, known as their homeostatic signature [53]. Components of the TGF-β signaling pathway such as Tgfbr1, Tgfbr1 and Smad3 are identified as homeostatic microglial genes [41]. Recent studies using the Cre-Lox system to selectively target components of the TGF-β signaling pathway have demonstrated its importance in maintaining the homeostasis and quiescence of adult microglia [2]. However, targeting different components with various promoters yields differing results. The tamoxifen-induced deletion of Tgfbr2 in adult Sal1^CreERT2^ mice (*Sall1*^CreER^:*Tgfbr2*^fl/fl^) results in microglia adopting a reactive morphology, characterized by shorter, thicker dendrites and enhanced immunoreactivity to the ionized calcium-binding adapter molecule 1 (Iba-1) [52]. This is accompanied by the upregulation of CD45, F4/80, MHCII and certain inflammatory cytokines, including Il1b, TNF and Cxcl10, similar to the microglia isolated from lipopolysaccharide (LPS)-challenged mice, which serve as a positive control for reactive microglia. The deletion of Tgfbr2 in *Cx3cr1*^CreERT2^ mice (*Cx3cr1*^CreER^:*Tgfbr2*^fl/fl^) in adult microglia results in a reactive microglia phenotype, evidenced by morphology and the increased expression of reactive markers [61,62]. However, the loss in TGF-β signaling does not affect postnatal survival or impair the expression of microglia-specific molecular signature genes such as P2ry12, Tmem119, Hexb and Sall1 [62] (Figure 2). Instead, it leads to the overexpression of markers associated with immature microglia [61]. Microglia-specific knockout of the Tgfb1 gene (*Cx3cr1*^CreER^:*Tgfb1*^fl/fl^ mice) results in transcriptomic changes indicative of disrupted homeostasis [54,63], closely resembling those observed in disease-associated, injury-associated and aged microglia [54]. Together, these findings suggest that constant basal TGF-β signaling is necessary for microglial homeostasis, which is vital to prevent excessive microglia activation under physiological conditions. Additionally, dysregulated neurogenesis [52] and cognitive deficits [54,63] have been observed in these mice, suggesting that the loss of TGF-β signaling in microglia could contribute to neuronal dysfunction and cognitive deficits.

Recent studies have shown that microglia secrete TGF-β1 to maintain their own homeostatic state in an autocrine manner. Microglia secrete TGF-β1 more than neurons or astrocytes in the adult brain [54] and express high levels of Tgfbr1 [53]. Consequently, astrocyte-specific or forebrain neuronal-specific Tgfb1 gene deletion does not affect the homeostasis of microglia [54]. Moreover, the depletion of microglia causes a reduction in TGF-β levels and myelin degeneration in the white matter, leading to cognitive impairment [64]. This further supports that microglia are a primary source of TGF-β1 and underscores their significance in maintaining proper brain function. Furthermore, TGF-β1 produced by microglia is tightly regulated in terms of its localization, activation and downstream signaling. The negative regulator of reactive oxygen species (NRROS), also known as LRRC33, anchors latent TGF-β onto the cell surface, which is essential for the activation of TGF-β1 in microglia. As a milieu molecule, it facilitates the highly localized, integrin-αVβ8-dependent activation of latent TGF-β. Mice deficient in Lrrc33 develop paraparesis and neurodegeneration and have reactive microglia [65]. Patients with biallelic NRROS mutations develop early-onset lethal microgliopathy [66], mirroring the clinical features observed in mice deficient in Nrros/Lrrc33. Together, these findings support that microglia are the primary producers of the TGF-β1 ligands necessary for their own homeostasis. Since TGF-β1 is mainly produced by homeostatic microglia in the CNS and microglial homeostasis is compromised with aging and neurodegenerative diseases, this points to a potential mechanism through which microglial dysfunction may contribute to the functional deficits observed in these conditions.

## 6. TGF-β Signaling in Microglia Reactivity

The influence of TGF-β on microglial homeostasis extends to its role in modulating the response to CNS injury and disease. The effects of TGF-β on microglial reactivity are highly context-dependent, influenced by factors such as the nature and duration of the CNS insult, the presence of other cytokines and the overall state of the neural environment.

The expression of homeostatic genes declines with aging, a characteristic feature of aged microglia [41]. TGF-β signaling is part of the aged human microglial signature, with genes in the TGF-β signaling pathway being downregulated [67]. Given its role in maintaining microglial homeostasis, it is reasonable to hypothesize that disrupted TGF-β signaling during aging promotes the loss of homeostasis in aged microglia and contributes to the pathophysiology of neurodegenerative diseases [68]. A recent scRNA-seq study lends partial support to this hypothesis. Dynamics and pseudo-time analyses identified intermediate states of microglial aging, revealing that TGF-β signaling can significantly impact microglial states [63]. This suggests that TGF-β signaling in microglia plays a crucial role in shaping microglial aging trajectories.

LDAM (characterized by lipid droplet accumulation) is a prominent phenotype observed in aged and diseased microglia [36]. In LDAM, the TGF-β signaling pathway is downregulated, driving microglia towards a pro-inflammatory phenotype [69] (Figure 2). In primary microglia culture, elevating TGF-β1 expression suppresses inflammation and reduces lipid droplet accumulation when the cells are exposed to LPS. Conversely, the inhibition of TGF-β1 leads to microglial inflammation and greater lipid droplet accumulation in microglia exposed to oxygen–glucose deprivation injury [70].

Cre-Lox-based mouse lines targeting components of the TGF-β pathway offer avenues for analyzing the role of this pathway not only in microglial homeostasis (in the absence of disease stimuli) but also in microglial reactivity within disease contexts. In the adult *Cx3cr1*^CreER^:*Tgfbr2*^fl/fl^ mice, constitutive TGFBR2 expression in retinal microglia is specifically ablated following tamoxifen administration [71]. This ablation induces rapid morphological transformation, proliferation and the heightened reactivity of microglia, leading to secondary Müller cell gliosis, neuronal degeneration and decreased light-evoked retinal function. These findings indicate that deficiencies in microglial TGF-β signaling can drive neuroinflammatory contributions to retinal neurodegeneration, highlighting TGF-β signaling as a potential therapeutic target. In support of this, the AAV-mediated delivery of TGF-β1 rescues degenerating cones and preserves vision in the mouse models of retinitis pigmentosa [72]. These protective effects are mediated by microglia, as they are disrupted by either depleting microglia or the blocking of the TGF-β receptors.

The *Cx3cr1*^CreER^:*Tgfbr2*^fl/fl^ mice have also been used to study the integration of peripheral monocyte-derived macrophages into the microglia niche in microglial depletion models. When bone marrow cells from *Cx3cr1*^CreER^:*Tgfbr2*^fl/fl^ mice are transferred into Cx3cr1^CreER^:R26^DTA^ mice after microglia are depleted [73], Tgfbr2^−/−^ monocytes cross the BBB and integrate into the microglia niche while displaying an inflammatory profile. The recipient animals develop rapidly progressive and fatal demyelinating motor disease, culminating in complete paralysis and premature death. Notably, the presence of adaptive immune cells is not observed in the recipient animals, indicating that Tgfbr2^−/−^ macrophages directly induce demyelination and neuronal damage.

By selectively targeting microglial TGF-β signaling, these studies emphasize its importance under disease conditions. However, these and similar mouse models have not yet been integrated with common neurodegenerative disease models. Such research could enhance our understanding of the TGF-β-mediated regulation of microglial reactivity in neurodegenerative diseases, potentially paving the way for new therapeutic approaches.

While TGF-β signaling regulates microglial reactivity, the dysfunction of microglia can, in turn, impact TGF-β signaling. The loss of the fractalkine receptor (CX3CR1), a homeostatic gene in microglia, in 5xFAD transgenic mice, led to microglial dysregulation and heightened reactivity, resulting in impaired Aβ phagocytosis and clearance, along with aberrant TGF-β signaling [74].

## 7. TGF-β1 Signaling in Neurological Diseases: Implications for Treatment

Since aging is the primary risk factor for the development of neurodegenerative disease [75], the downregulation of the TGF-β signaling pathway and the loss of homeostasis in aged microglia likely contribute disease progression. In AD, despite conflicting findings regarding TGF-β1 levels at various stages, TGF-β signaling is decreased in the brain [13] and CSF [76]. TGF-β1 has emerged as one of the leading CSF candidate biomarkers associated with the rate of cognitive decline in dementia patients [77]. Thus, the restoration of impaired TGF-β signaling might represent a promising pharmacological strategy for attenuating neurodegenerative diseases [78]. The intracerebral delivery of a viral construct expressing a constitutively active form of Tgfbr1, which activates TGF-β signaling without the need for ligands, reduced gliosis and rescued dopaminergic neurons in the MPTP model of PD [79]. In a mouse model of AD, the intracerebroventricular injection of TGF-β1 prevented hippocampal dendritic spine loss and memory impairment in the mice that received an intracerebroventricular infusion of amyloid-β oligomers [80].

The soluble form of TGF-β receptor 3 (sTGFBR3) is generated through the proteolytic cleavage of TGFBR3, which is predominantly expressed on the surface of microglia. While TGFBR3 facilitates the presentation of TGF-β ligands to TGFBR2, thus initiating TGF-β signaling, sTGFBR3 can competitively bind to TGF-β ligands, thereby preventing the activation of the TGF-β pathway. In the APP/PS1 mouse model of AD, the augmentation of TGF-β signaling by knocking down Tgfbr3 suppressed the NF-κB pathway, leading to a reduction in the number of DAMs, the inhibition of their pro-inflammatory activity and the restoration of spatial learning and memory [81]. The intramuscular administration of plasmids encoding TGF-β polynucleotides enhanced the spatial memory performance of and reduced the neuroinflammation in the Tg-2576 mouse model of AD [82].

In the acute phase of traumatic brain injury (TBI) and stroke, TGF-β signaling is typically activated due to increased levels of TGF-β1 and its receptors [5]. Notably, enhancing TGF-β signaling in these situations has been found to be protective. For instance, treatment with TGF-β1 via intracerebroventricular injections leads to improved functional recovery following TBI [83] and intracerebral hemorrhage [84]. Similarly, the activation of the TGF-β signaling pathway after stab injury restrains inflammation and boosts the tissue reparative responses of reactive astrocytes and microglia [85]. As in AD and PD studies, these studies often report reduced microglia reactivity, which is believed to be a primary contributor to the protective effects of TGF-β. However, it should be noted that delivering TGF-β ligands to the brain can activate TGF-β signaling in other cell types, which may also contribute to the observed protective effects. Nevertheless, these studies support that the augmentation of TGF-β signaling exerts protective effects across multiple models of neurological disorders [78].

Contrary to the findings that the activation of TGF-β signaling enhances microglial reparative responses following TBI [85], its activation has been shown to impair microglial reparative innate immune responses in a mouse model of demyelinating injury [86]. This model required microglial innate immune function for a regenerative response to occur. The activation of the TGF-β signaling pathway after a Western diet suppresses the microglial liver X receptor pathway, inhibiting phagocytic clearance of myelin and cholesterol, thereby impairing lesion recovery after demyelination. Blocking TGF-β restores microglial responsiveness and myelin–debris clearance after demyelinating injury [86]. This further supports the context-dependent role of TGF-β signaling in microglial function.

In recent years, numerous studies have highlighted the neurobiological and clinical connections between depression and AD [87]. Depression is a significant risk factor for developing AD [88] and the presence of depressive symptoms increases the risk of the proximal development of Mild Cognitive Impairment (MCI) due to AD [89]. Several studies in patients with Major Depressive Disorder (MDD) have found that their plasma TGF-β1 levels were reduced and correlated with the severity of their depression [87]. Lower TGF-β1 levels may significantly contribute to treatment resistance in MDD. Genetic variation in TGFBR1 influences the association between amygdala volume and prenatal depressive symptoms [90]. Given that deficits in TGF-β signaling contribute to the pathogenesis of AD and cognitive decline, these findings suggest that TGF-β signaling could serve as a common pharmacological target for both depression and AD [87]. Indeed, treatment with antidepressants alleviates depression-related behaviors and memory impairments in mice injected with Aβ, primarily by restoring hippocampal TGF-β1 levels [91]. Notably, a novel microglial TGF-β1-dependent mechanism has been discovered that underlies the antidepressant effects of (R)-ketamine in rodent models of depression [92]. Antidepressants may be potential candidates for reducing microglial reactivity in neurodegenerative diseases [93]. Together, these studies suggest that the potential restoration of TGF-β signaling via antidepressants may offer a promising strategy for treating AD.

## 8. TGF-β Signaling in APOE4-Mediated Microglial Dysfunction

Recently, the triggering receptor expressed on the myeloid cells 2 (TREM2)–Apolipoprotein E (APOE) pathway has been indicated to drive the functional switch of microglia from a homeostatic to a DAM/MGnD phenotype [32,34]. Importantly, this MGnD phenotype is regulated by the TGF-β pathway [34]. The activation of the TREM2-APOE pathway is a key transcriptional signature of reactive microglia, conserved in multiple mouse models of neurodegenerative diseases such as AD (APP/PS1 mice), amyotrophic lateral sclerosis (ALS) (SOD1G93A mice) and multiple sclerosis (MS) (experimental autoimmune encephalomyelitis) [34]. The TREM2-APOE pathway has also been found in transcriptomic post-mortem analyses of human brains, with APOE4 carriers exhibiting a more prominent pro-inflammatory and phagocytic microglial transcriptomic signature [94].

Apolipoprotein E4 (APOE4) is the strongest genetic risk factor for early and late onset-AD [95]. Studies from AD patients, patient-derived stem cell models and human APOE4-expressing and APOE-deficient mouse models have demonstrated that APOE4 provokes neuroinflammation, impairs vascular function and exacerbates Aβ and tau pathology [96]. One mechanism by which APOE4 influences disease outcome is through the regulation of microglial function and reactivity [95]. As a key lipid transporter between neurons and glial cells, APOE plays a significant role in microglial metabolism and lipid accumulation [95]. A lipidome analysis of microglia that was isolated from mice expressing human APOE variants revealed aberrant lipid metabolism in APOE4 microglia [33]. Homeostatic microglia primarily depend on oxidative phosphorylation for energy production, whereas reactive microglia with elevated APOE4 expression shift toward glycolysis and exhibit the upregulation of hypoxia-inducible factor 1-alpha (HIF1α) [97]. APOE4 promotes lipid droplet accumulation in LDAM. APOE4 hiPSC-derived microglia (hiMGL) accumulate more lipid droplets than APOE3 microglia [98]. Similarly, the selective expression of human APOE4 in microglia in the APP/PS1 amyloidosis model leads to increased lipid accumulation in APOE4 microglia compared to those expressing APOE3 [99].

APOE4 microglia exhibit a reactive phenotype characterized by exacerbated pro-inflammatory responses, along with dysregulated phagocytosis and chemotaxis [95]. This heightened reactivity is thought to contribute to the progression of neurodegenerative diseases by exacerbating neuroinflammation and neuronal damage. The selective removal of microglial APOE4 in APP/PS1 mice leads to a significant reduction in plaque loads [33], suggesting a ‘gain-of-toxic’ effect of APOE4 on microglia-mediated Aβ clearance. Conversely, the selective expression of APOE3 in microglia in APP/PS1 mice provides sufficient protection against the development of amyloid pathology, whereas APOE4 microglia fail to do so [99]. The defective response of APOE4 microglia to Aβ pathology appears to be mediated by the TGF-β pathway [33]. When human APOE3 or APOE4 was specifically expressed in the microglia of the P301S tauopathy model, APOE3-expressing microglia exhibited the neurodegenerative phenotype MGnD, accompanied by a downregulation of homeostatic transcripts such as Tgfb1 and Smad3. In contrast, APOE4-expressing microglia displayed a blunted MGnD response and increased TGF-β signaling. Notably, the deletion of microglial APOE4 reversed many of these transcriptional deficiencies, reducing tau hyperphosphorylation and preventing neuronal loss. Similar findings were observed with human APOE3 or APOE4 specifically expressed in the microglia of APP/PS1 mice [33]. The mice with APOE4 microglia had more Aβ plaque formation and less microglia surrounding the plaques compared to the mice expressing APOE3 in their microglia. The deletion of microglial APOE4 resulted in reduced Aβ pathology and increased microglial recruitment to plaques. Transcriptionally, the APOE3 microglia readily adopted an MGnD signature, with a high expression of Lgals3 and Clec7a, while APOE4-expressing microglia showed an impaired MGnD response driven by the upregulation of homeostatic checkpoint genes such as ITGB8 and Inpp5d. The microglial-specific deletion of APOE4 restored the MGnD signature, increasing plaque-associated microglia and plaque compaction and reducing both Aβ pathology and dystrophic neurites. The downregulation of MGnD-associated genes as well as increased TGF-β signaling were also observed in the human APOE4 carriers. Importantly, the reduction in ITGB8 signaling via genetic knockout or a neutralizing antibody enhanced microglial Aβ phagocytosis, with the upregulation of antigen presentation and interferon genes [33]. Taken together, these studies support the beneficial role of MGnD in limiting AD pathology and protecting against cognitive decline. This protective phenotype is notably impaired in APOE4 carriers and is critically dependent on the suppression of TGF-β signaling. In summary, these studies show that microglial ApoE3 induces MGnD genes for plaque encapsulation and clearance, while microglial ApoE4-induced ITGB8-TGF-β signaling impairs this MGnD response, thereby exacerbating amyloid pathology. The findings that APOE4 impairs the microglial response in AD by inducing TGF-β-mediated checkpoints strongly support a mechanistic link between APOE4 and aberrant TGF-β signaling in the disease process. In the context of APOE4 being the strongest genetic risk factor for AD, increased odds ratios have been observed in female carriers [100]. TGF-β signaling is involved in sex-dependent APOE4 neutrophil–microglia interactions, which drive cognitive impairment in AD [101]. In particular, APOE4 female IL-17+ neutrophils upregulate TGF-β and importantly, SMAD2, a downstream effector of TGF-β signaling, is a core transcriptional regulator underlying sex difference.

The seemingly paradoxical findings that both downregulated and heightened TGF-β signaling can have detrimental effects—promoting LDAM (a pro-inflammatory and neurotoxic phenotype) with downregulated TGF-β signaling and impairing MGnD (a protective phenotype) with heightened TGF-β signaling—underscore the complex, context-dependent nature of TGF-β signaling, even within a single cell type. Therefore, both excessive and insufficient TGF-β signaling can be detrimental, highlighting the importance of maintaining balanced TGF-β signaling for brain homeostasis. This has been demonstrated in myelin maintenance and remyelination, where both excessive and insufficient TGF-β signaling are detrimental to myelin health [102]. These observations further suggest that the dynamic and precise regulation of TGF-β signaling in response to aging- and neurodegeneration-associated pathologies is necessary for fine-tuning microglial responses, emphasizing the need for targeted therapeutic strategies that consider the timing and context of its application.

TREM2 is also closely linked to TGF-β signaling in microglia. TGFBR2 modulates the levels of soluble TREM2 (sTREM2) in cerebrospinal fluid (CSF). In cell-based assays, the overexpression or knockdown of TGFBR2 leads to significant changes in sTREM2 levels [103]. On the other hand, TREM2 deficiency leads to the dysregulation of TGF-β signaling, which triggers abnormal inflammatory responses and apoptosis. In a mouse model of ischemic stroke, TREM2 knockdown reduced TGF-β1 expression and decreased the number of anti-inflammatory phenotype microglia, leading to increased cerebral infarct size, exacerbated neuronal apoptosis and aggravated neuronal impairment [69]. These findings reveal a regulatory feedback loop between TREM2 and TGF-β1 that helps maintain microglia homeostasis and limit inflammation in the CNS.

## 9. Discussion, Conclusions and Future Directions

Microglia have emerged as the therapeutic targets for a wide range of CNS disorders and diseases [1]. Current microglia-related therapies have shifted from simply attenuating excessive microglial reactivity and phagocytic activities to targeting the complex and context-dependent heterogeneity of microglial states [104]. Therapies attempting to target specific state-associated microglial properties and functions are currently under investigation. TGF-β signaling presents a promising opportunity for such therapeutic interventions.

As a pleiotropic cytokine, TGF-β plays a crucial role in regulating multiple aspects of microglial biology, from their development and maintenance of homeostasis to their response to injury and disease. As the resident immune cells of the CNS, microglia rely on a finely tuned balance between homeostasis and reactivity, with TGF-β being essential for maintaining this equilibrium across various functional states. A deeper understanding of how TGF-β influences microglial function offers significant potential for advancing our knowledge of CNS health and disease, while also opening new avenues for therapeutic interventions aimed at preventing or mitigating neurodegenerative diseases driven by microglial dysfunction [13]. As research continues to unravel this complex signaling pathway, several key areas are emerging as promising directions for further investigation.

One area of focus is understanding when microglial functions become maladaptive and the role TGF-β signaling plays in this process. TGF-β’s involvement in microglial reactivity is highly complex and context-dependent. Microglia can adopt a range of reactive states, from adaptive (neuroprotective) to maladaptive (neurotoxic). TGF-β signaling can suppress excessive inflammation, thereby protecting neural tissue, or contribute to pathological states if its regulation is disrupted. A deeper understanding of these dynamics is crucial for developing targeted therapies that modulate TGF-β signaling to maintain microglial homeostasis while preventing excessive reactivity. When aiming to treat different neurologic diseases, a fine-tuned approach to modulating TGF-β signaling—enhancing its protective effects while minimizing potential harmful off-target effects—remains a compelling yet challenging strategy [60]. Future efforts should prioritize understanding the context-dependent effects of TGF-β on microglia and fine-tuning the balance between adaptive and maladaptive states to mitigate neuroinflammatory responses in CNS disorders.

Another promising direction is the study of TGF-β‘s role in age-related changes in microglial function. As the brain ages, the regulation of microglial activity by TGF-β may shift, contributing to increased vulnerability to neurodegenerative conditions. Investigating these age-related dynamics could lead to strategies for maintaining microglial health throughout aging. Furthermore, advancing our understanding of how genetic variations in TGF-β signaling components influence microglial behavior may provide insights into individual susceptibilities to CNS diseases.

The BBB presents a challenge for delivering therapeutics targeting TGF-β signaling in the brain. There is an urgent need for the development of strategies to overcome this barrier [78]. We recently developed a novel small molecule TGF-β activator, C381, that is capable of penetrating the brain [105]. C381 treatment results in a dose-dependent reduction in microgliosis in Progranulin^−/−^ mice, a model of lysosomal storage disease and frontotemporal dementia [105]. The effects of C381 treatment on microgliosis are also independently observed in mice with experimental autoimmune encephalomyelitis (EAE), a model of multiple sclerosis [106]. Therefore, C381 is a first-in-class small molecule TGF-β activator capable of modulating microglial reactivity and holds significant potential for treating neurodegenerative disorders.

In conclusion, future research on TGF-β in regulating microglial homeostasis and reactivity offers exciting opportunities to develop novel therapeutic strategies aimed at preventing or treating a wide range of CNS disorders. By deepening our understanding of TGF-β‘s roles in microglia, we can move closer to harnessing its full potential for maintaining brain health and combating disease.

## Data Availability

Not applicable.
